# Ultrafast Multi-Level Logic Gates with Spin-Valley Coupled Polarization Anisotropy in Monolayer MoS_2_

**DOI:** 10.1038/srep08289

**Published:** 2015-02-06

**Authors:** Yu-Ting Wang, Chih-Wei Luo, Atsushi Yabushita, Kaung-Hsiung Wu, Takayoshi Kobayashi, Chang-Hsiao Chen, Lain-Jong Li

**Affiliations:** 1 Department of Electrophysics, National Chiao Tung University, Hsinchu, Taiwan; 2 Taiwan Consortium of Emergent Crystalline Materials, Ministry of Science and Technology, Taipei 10601, Taiwan; 3 Advanced Ultrafast Laser Research Center and Department of Engineering Science, The University of Electro-Communications, Chofu, Tokyo 182-8585, Japan; 4 JST, CREST, 5 Sanbancho, Chiyoda-Ku, Tokyo 102-0075, Japan; 5 Center for Micro/Nano Science and Technology, National Cheng Kung University, Tainan, Taiwan; 6 Institute of Atomic and Molecular Science, Academia Sinica, Taipei, Taiwan

## Abstract

The inherent valley-contrasting optical selection rules for interband transitions at the K and K′ valleys in monolayer MoS_2_ have attracted extensive interest. Carriers in these two valleys can be selectively excited by circularly polarized optical fields. The comprehensive dynamics of spin valley coupled polarization and polarized exciton are completely resolved in this work. Here, we present a systematic study of the ultrafast dynamics of monolayer MoS_2_ including spin randomization, exciton dissociation, free carrier relaxation, and electron-hole recombination by helicity- and photon energy-resolved transient spectroscopy. The time constants for these processes are 60 fs, 1 ps, 25 ps, and ~300 ps, respectively. The ultrafast dynamics of spin polarization, valley population, and exciton dissociation provides the desired information about the mechanism of radiationless transitions in various applications of 2D transition metal dichalcogenides. For example, spin valley coupled polarization provides a promising way to build optically selective-driven ultrafast valleytronics at room temperature. Therefore, a full understanding of the ultrafast dynamics in MoS_2_ is expected to provide important fundamental and technological perspectives.

Structural inversion symmetry together with time reversal symmetry allows monolayer MoS_2_ to possess the same magnitude of magnetic moments but the opposite signs at the K and K′ valleys^1^,^2^. Furthermore, spin-orbit coupling separates the spin-up and spin-down states of the valence band^3^,^4^,^5^, which plays a crucial role in spintronics^6^, valleytronics^7^, and semiconductor devices^8^,^9^. As shown in Figure 1(a), there is fascinating coupling between energy, spin, and valley. Utilizing these degrees of freedom of monolayer MoS_2_, optically driven logic gates can be realized. Figure 1(b) illustrates that a two-level logic gate can be operated by being sequentially excited with circularly polarized 2.01 eV (resonant with exciton B) and 1.98 eV (resonant with exciton A) pulses at room temperature. According to our time-resolved studies, the nonequilibrium population between the K and K′ valleys lasts for ~1 ps in monolayer MoS_2_, which is an excellent candidate material for the ultrafast optical control. For the application to a high-rate optical pulse control, the problem of the accumulation of the remnant coherence after the control pulse always exists. Thus, the following pulse to control the succeeding step must wait for the decoherence of the target. This limits the bandwidth of optical spin controlling devices.

Circularly polarized luminescence from monolayer MoS_2_ has been demonstrated to have the same helicity as the circular polarization of an excitation laser^3^,^4^,^5^,^10^,^11^. This highly polarized luminescence has only been observed with resonant excitation. Furthermore, the valley-spin lifetime was previously predicted to be >1 ns^3^. However, a time-resolved study of polarized photoluminescence (PL) exhibited that the carrier spin flip time is in the time scale of several picoseconds, which is limited by the time resolution of the PL measurement system^12^. Mai et al.^13^ further observed that the polarized exciton A decays only within several hundred femtoseconds, according to optical pump-probe measurements. Under the controversial situation, the full dynamics and physical insight of the polarized excitons in monolayer MoS_2_, including the spin-valley coupling, have not yet been conclusively studied.

In this work, we propose optically driven ultrafast two-level MoS_2_-logic gates through a systematic study of the ultrafast dynamics of monolayer MoS_2_ with 30-fs time resolution. The identification of a single atomic layer of MoS_2_ is confirmed by photoluminescence spectra, Raman spectra and AFM measurements, as shown in Figure 2. Through the resonant and off-resonant excitations in the direct bandgap, the valley polarization dynamics in monolayer MoS_2_ was clearly observed. The pump pulse used in this study was generated by a wavelength-tunable optical parametric amplifier. Meanwhile, a probe pulse with a visible broadband spectrum was produced by self-phase modulation in a sapphire plate (see Supplementary Information, S4). As shown in Figure 3, the absorption spectrum of a monolayer MoS_2_ clearly presents A (~1.89 eV) and B (~2.04 eV) excitonic transitions, which indicates the splitting of the valence band at the K valley due to spin-orbit coupling^14^,^15^. In order to distinguish the non-equivalent K and K′ valleys, polarizations of pump and probe pulses were adjusted to be circularly polarized by quarter wave plates.

Figure 4 shows a 2D display of photon energy- and time-resolved transient difference absorbance Δ*A*(*ω*, *t*) at 78 K. In the measurements, the polarizations of broadband probe pulses were adjusted to be *σ*^+^ and *σ*^−^ while the pump pulses were set as *σ*^+^ circular polarization and 1.89 eV to resonate with exciton A. For both *σ*^+^ and *σ*^−^ probes, the time-resolved spectra exhibited negative Δ*A* in the spectral band of exciton A and exciton B. The negative Δ*A* signal could be caused by stimulated emission from the excited state and/or photobleaching due to the depletion of the ground state and the population of the excited state. The lifetime of photobleaching is usually much longer than that of stimulated emission because a stimulated emission occurs only within the lifetime of the excited state, whereas photobleaching occurs until the ground state is fully repopulated. Obviously, Δ*A* is significantly probe polarization-dependent within 100 fs as shown in Figure 4(c) and Figure 4(d). The nonlinear optical response after 100 fs is independent of the angular momentum of the initially excited distribution, which indicates that the initial polarization distribution relaxes to some quasi-equilibrium states in 100 fs. Following the fast spin-polarization relaxation, the peaks in the Δ*A* spectrum show a blue-shift before 10 ps and a red-shift after 10 ps as shown in Figure 4(a) and Figure 4(b). We note that the pump induced response at the K′ valley is observed even when exciton A at the K valley is excited by the *σ*^+^ pump, in contrast to the cases of excitons coupled to pump and probe pulses, which do not share common states. This unexpected phenomenon could be explained through various possible mechanisms, e.g., the dark excitons generated by pump pulses^13^, the weakening of the excitonic binding energy^16^ or the dielectric screening from the excited excitons^16^.

The measured time-resolved traces of Δ*A*(*ω*, *t*) were fitted using the sum of three exponential functions and a constant term, as in equation (1).
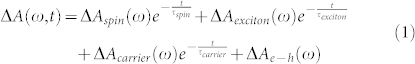
The fitting results are shown in Figure 5. For the *σ*^+^ probe, the time constants, *τ_spin_*, *τ_exciton_* and *τ_carrier_* are 55 ± 7 fs, 1.0 ± 0.2 ps and 26.3 ± 5.4 ps, respectively. For the *σ*^−^ probe, they are 60 ± 40 fs, 0.96 ± 0.49 ps, and 25.7 ± 8.6 ps, respectively. Due to the small signal amplitudes at the photon energies of ~1.93 eV and ~2.08 eV, the fitting error is large. Comparing the spectra of the *σ*^+^ and *σ*^−^ probes in Figure 5(a), Δ*A_exciton_*, Δ*A_carrier_* and Δ*A_e–h_* exhibit similar dependence on the probe photon energy with the exception of Δ*A_spin_*. Thus, these three relaxation processes take place independently of the initial polarization distribution within 100 fs. However, Δ*A_spin_* is completely different from the cases of *σ*^+^ and *σ*^−^ probes in that it is dependent on the relative polarization between the probe and pump beams. In the case of the *σ*^+^ pump and *σ*^+^ probe, Δ*A_spin_* is negative and possesses larger amplitude than in the case of the *σ*^+^ pump and *σ*^−^ probe. This implies that the polarized exciton A at the K valley excited by the *σ*^+^ pump pulses leads to intense photobleaching and stimulated emission only when the probe pulses have the common circular polarization to the pump pulses and the probe photon energy overlaps the band of exciton A. Moreover, the spectral shape of Δ*A* fits well with excitonic transition A, which indicates that the valley polarization is efficiently excited at the high-symmetry K point^17^. On the other hand, the *σ*^−^ probe pulses generate exciton A with opposite spin polarization at the K′ valley. Excitons with opposite polarization leads to the generation of biexcitons, which is the origin of induced absorption (Δ*A* > 0) at ~1.87 eV^13^,^18^, as shown in the right panel of Figure 5(a).

The mean transition energy of excitonic band A is further calculated at every time delay (see Supplementary Information, S2), i.e., the time-dependent energy gap between excited electrons and holes, is plotted in the inset of Figure 5(a). Since the photobleaching (caused by the same polarizations *σ*^+^ for the pump and probe) and biexciton formation (caused by opposite polarizations *σ*^+^ for pump and *σ*^−^ for probe), the results of the *σ*^+^ and *σ*^−^ probes before 100 fs show distinct energy differences in transition A. After randomization of the polarized excitons generated by a pump pulse, the energy difference disappears. Blue shift and red shift take place when the polarizations of the pump and probe are the same and opposite, respectively. Similar energy difference (~13 meV at *t* ~ 0 ps in this study) is also observed on the CdSe nanocrystals with the splitting of bright-dark exciton states^19^,^20^. It is found that the flipping transition time scales with the energy split of bright-dark exciton states. Once the energy split increases to ~14 meV for the small size of nanocrystals, the flipping transition time is in the range of tens of femtoseconds. This implies that the energy difference between the K and K′ valleys caused by the inhomogeneity of initially excited population in the K and K′ valleys leads to the fast spin randomization time. Moreover, the exciton size (diameter ~ 1.86 nm^21^) and spin randomization time (~60 fs) of monolayer MoS_2_ also satisfy the size dependence of spin flip rate in semiconductor nanocrystals^19^,^20^. The behaviors can be rephrased as follows. The time constant *τ_spin_* ~ 60 fs reflects the lifetime of spin polarized exciton A. It has relevant decay time but different behaviors between the co-circular polarizations and anti-circular polarizations of the pump and probe beams. Since the hole spin states are non-degenerated at the K and K′ valleys, the relaxation of hole spin is blocked by spin-valley coupling. On the contrary, spin states of electrons can be easily destroyed because the spin states of the conduction band are degenerate. Thus, the relaxation of the electron spin of excitons causes the transition from optically active excitons to dark excitons.

The difference absorbance, Δ*A_exciton_*, probed by *σ*^+^ and *σ*^−^ circular polarizations have the same photobleaching phenomena, i.e., the blue shift of exciton A in the time range of <10 ps. This implies that the excess population of excitons is created in both the K and K′ valleys through intervalley scattering, which further reveals the relaxation of hole spin polarization. Renormalization of the self-energy of the exciton is induced by mutual exciton-exciton interaction leading to photobleaching together with a blue shift of the exciton band within the exciton lifetime^22^. Therefore, Δ*A_exciton_* with a time constant of *τ_exciton_* ~ 1 ps represents the exciton intervalley transition and dissociation rate.

After the excitons are dissociated to become free carriers in highly excited states, the exciton peak exhibits a red shift. This shift is attributed to the intravalley scattering of free carriers, in which electrons relax to the bottom of the conduction band and holes relax to the top of the valence band. The Δ*A_carrier_* spectra show the sum of bleaching at transition energy peaks and the induced absorption of broad conduction band. Thus, the intermediate relaxation time *τ_carrier_* ~ 25 ps was assigned to the intraband transition of free carriers. The decay time of Δ*A_e–h_* is found to be too long to be determined in the present work. The constant term Δ*A_e–h_*, exhibits only bleaching behavior which can be attributed to the electron-hole recombination in the direct band. The recombination time was estimated to be ~300 ps in a previous study^23^.

Time-resolved measurements were also performed with the *σ*^+^ pump and *σ*^+^ probe at room temperature (293 K). The fitting results of the measured pump-probe data are shown in Figure 6. The amplitude of Δ*A* becomes lower and the peak positions shift to lower energy at 293 K compared with the result at 78 K. The red shift of Δ*A* implies the band gap is reduced with increasing temperatures^24^. The amplitude ratio of Δ*A_spin_* to Δ*A_exciton_* at different temperatures are more or less similar, i.e., 2.22 ± 0.27 for 78 K and 1.81 ± 0.49 for 293 K. This indicates that the spin-valley coupled polarizations are almost the same at various temperatures^12^.

The same experiment was also performed by changing the excitation energy from 1.89 eV to 2.01 eV to be resonant to the band of exciton B instead of exciton A (see Figure 3). Figure 5(c) shows the spectra of three relaxation components obtained from the fitting using equation (1). Δ*A_spin_* has finite size not only in the energy range of exciton B but also the energy range slightly higher than that of exciton A. This is because the excitation at 2.01 eV with a *σ*^+^ circular polarization simultaneously populates both exciton B at the K′ valley, resulting from the resonance excitation caused by the *σ*^+^ pump, and the higher energy side of exciton A at the K valley. Therefore, the calculated difference absorbance Δ*A_spin_* covers the band of transition B and the region of the high energy side of transition A. Additionally, Δ*A_exciton_* is comparable with Δ*A_spin_* in the energy range of exciton B and even larger than Δ*A_spin_* in the energy range of exciton A. Consequently, the signal of spin-valley coupled polarization can be sizable in the energy range of exciton B, which is the resonance excitation energy of the pump, as in the case shown in Figure 4(a), but its polarization anisotropy becomes much smaller. On the other hand, the spin-valley coupled polarization in the energy region of exciton A, which is off-resonant, is strongly diminished. This kind of reduction in spin-valley coupled polarization anisotropy, as observed in the PL experiments^11^,^12^, could be attributed to efficient intervalley scattering.

In conclusion, the present study completely elucidates the fairly comprehensive ultrafast dynamics of spin-polarized excitons in monolayer MoS_2_. Owing to the high temporal resolution and visible broadband detection, the time constants for the 60-fs spin-polarized exciton decay, 1-ps exciton dissociation (intervalley scattering), and 25-ps hot carrier relaxation (intravalley scattering) have been clearly identified. Temperature-dependent measurements further disclose the transition energy shifting and conservation of spin-valley coupled polarizations at various temperatures. Moreover, substantial intervalley scattering strongly diminished the spin-valley coupled polarizations under off-resonant excitation condition. These results provide a complete understanding of spin-valley coupled polarization anisotropy and the carrier dynamics of atomic layer MoS_2_, which can further help us to develop the ultrafast multi-level logic gates at room temperature.

## Author Contributions

C.W.L. conceived the research. Y.T.W., C.W.L., A.Y., K.H.W. and T.K. developed the concept and designed the experiments. Y.T.W. and A.Y. developed the experimental setup. Y.T.W. performed the helicity-resolved transient spectroscopy and prepared the manuscript. C.H.C. and L.J.L. synthesized and characterized the samples. All authors discussed the results and commented the manuscript.

## Supplementary Material

Supplementary Information Supplementary information

## Figures and Tables

**Figure 1:**
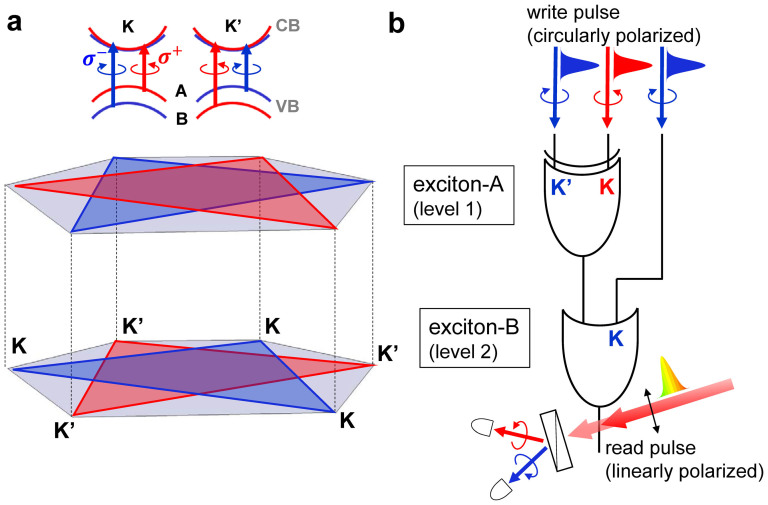
Schematics of an optically driven ultrafast room-temperature and multi-level logic gate with monolayer MoS_2_. (a) The band diagram of monolayer MoS_2_ at the K and K′ valleys. The blue and red colors represent spin-up and spin-down states, respectively. (b) A two-level MoS_2_-gate can be written by circularly polarized 2.01 eV (resonant with exciton B) and 1.98 eV (resonant with exciton A) pulses and read by a linearly polarized pulse with a visible broadband spectrum.

**Figure 2:**
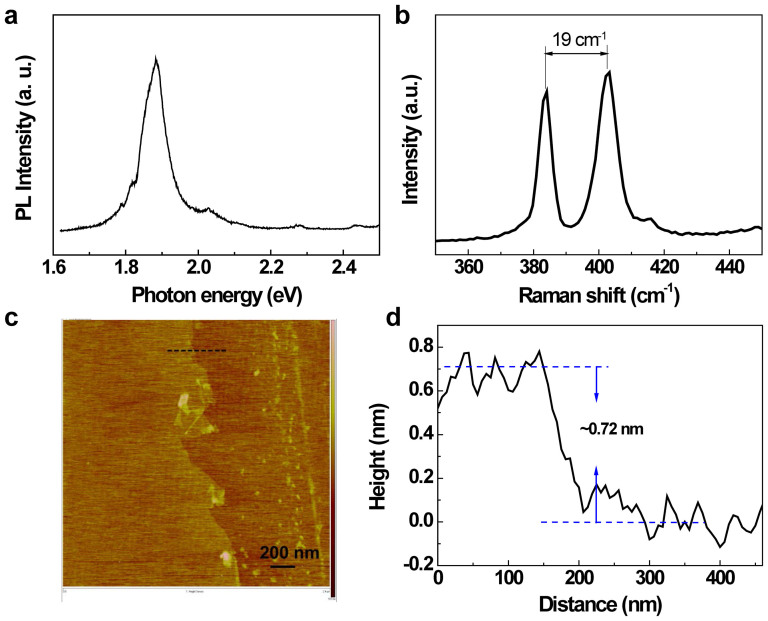
Characterization of monolayer MoS_2_. (a) A photoluminescence spectrum, (b) a Raman spectrum and (c) an AFM measurement of monolayer MoS_2_. (d) The height profile of MoS_2_ gives an average thickness of ~0.72 nm.

**Figure 3:**
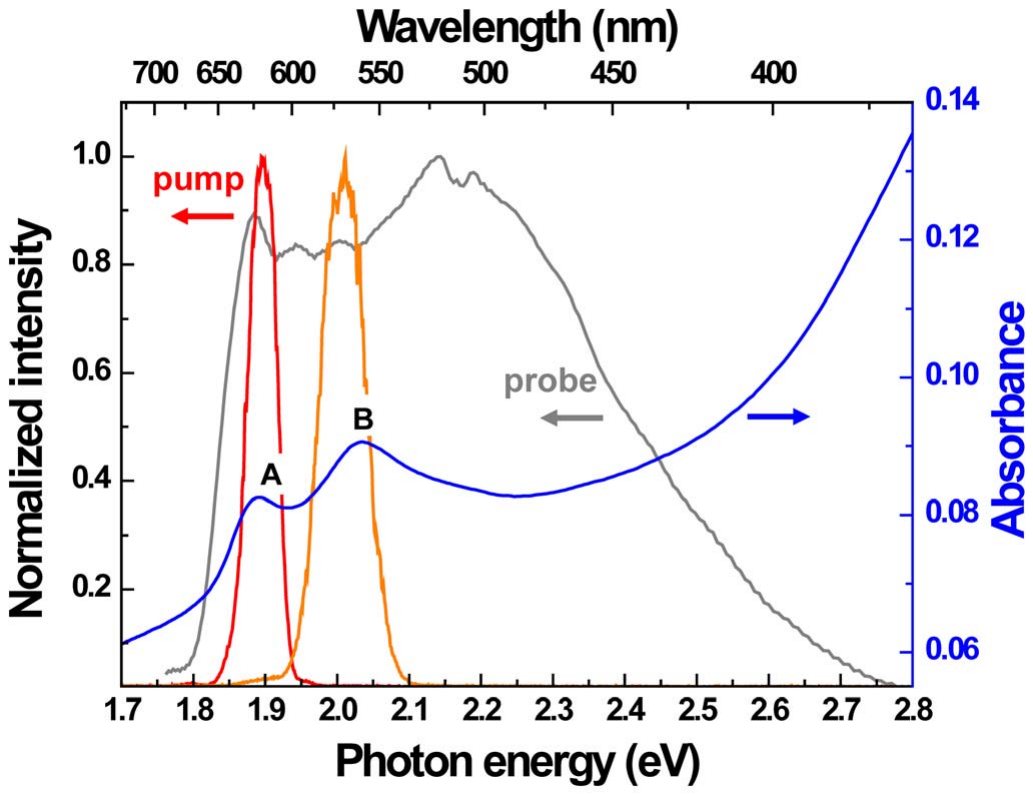
Spectra of pump, probe pulses and the stationary absorbance of monolayer MoS_2_. Spectra of 1.89 eV pump pulse (red), 2.01 eV pump pulse (orange), broadband visible probe pulse (gray) and the stationary absorption of monolayer MoS_2_ at room temperature (blue).

**Figure 4:**
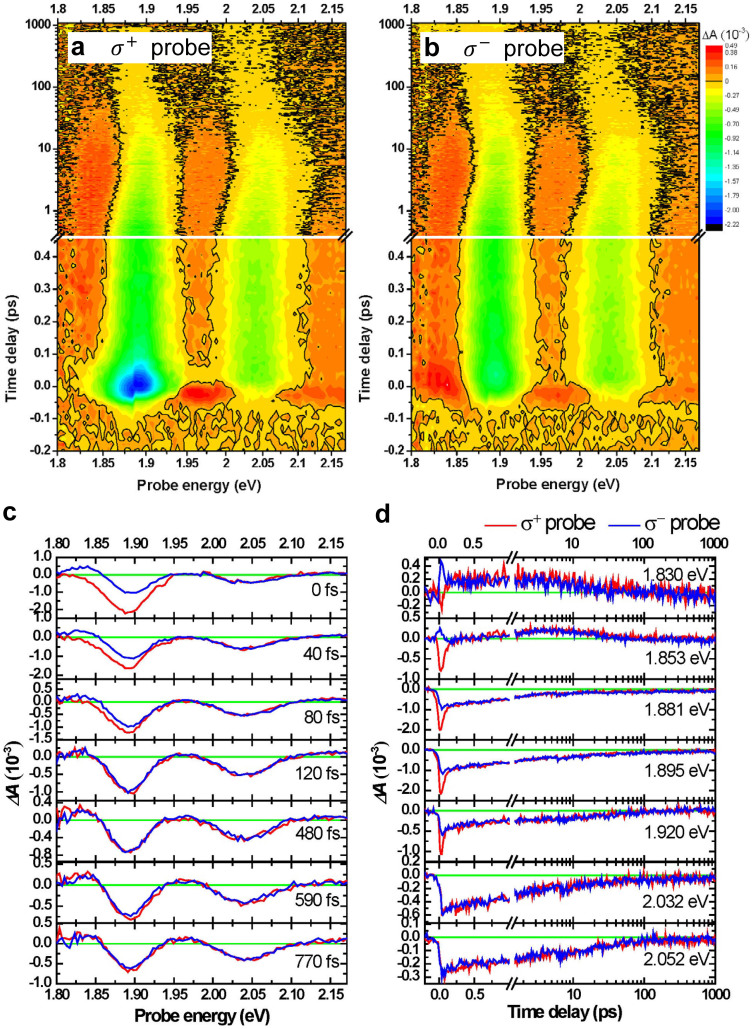
Transient difference absorbance (Δ*A*). Δ*A* induced by excitation using *σ*^+^ circularly polarized pump pulse with the photon energy of 1.89 eV and probed by (a) *σ*^+^ and (b) *σ*^−^ circularly polarized pulse at 78 K. The black curves are contours of Δ*A* being zero. (c) Time-resolved Δ*A* spectra at various time delays between pump and probe pulses. (d) Delay time traces of Δ*A* at various probe photon energies. In (c) and (d), the red and blue lines represent *σ*^+^ and *σ*^−^ probe, respectively. The horizontal green lines show Δ*A* = 0.

**Figure 5:**
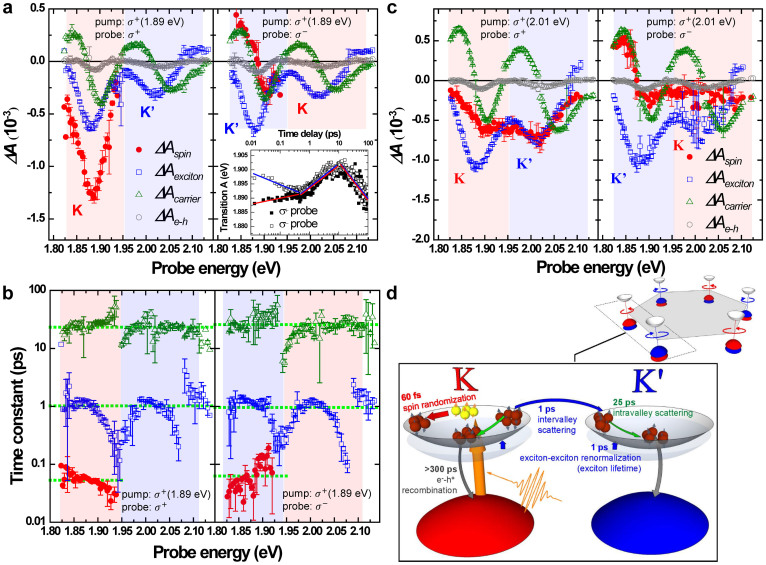
Triple exponential fitting results of the delay time traces of Δ*A* data at 78 K and the scheme of relaxation processes. (a), (b) Excited by 1.89 eV and *σ*^+^ pump pulse. (c) Excited by 2.01 eV and *σ*^+^ pump pulse. Left column: *σ*^+^ probe. Right column: *σ*^−^ probe. Solid circles (red), open squares (blue), open triangles (green), and open circles (gray) represent the components for spin randamization, exciton dissociation, hot carrier relaxation and electron-hole recombinataion, respectively. (a) and (c) Δ*A* spectra, (b) time constant of each component. Dot lines indicate the estimated values. Inset of (a): time-dependent (in log scale) mean energy of transition band A excited by 1.89 eV and *σ*^+^ pump pulse at 78 K. The solid squares are the *σ*^+^ probe and the open squares are the *σ*^−^ probe. (d) Schematics of the relaxation processes in monolayer MoS_2_.

**Figure 6:**
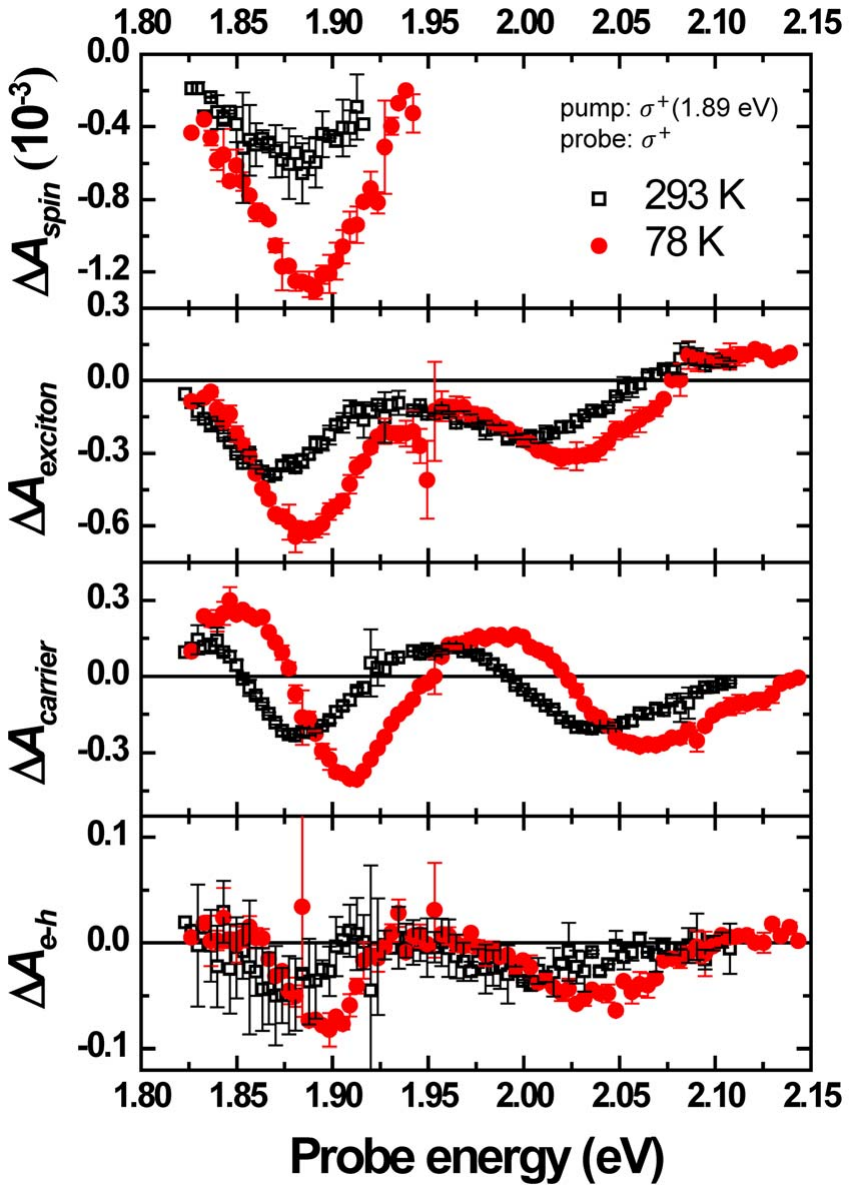
Comparison of the triple exponential fitting results at 78 K and 293 K. Triple exponential fitting results of the delay time traces of Δ*A* data excited by 1.89 eV with *σ*^+^ pump and *σ*^+^ probe pulse at 293 K (black open squares) and 78 K (red solid circles).
