# Supplementary material for: Ultrafast Multi-Level Logic Gates with Spin-Valley Coupled Polarization Anisotropy in Monolayer MoS2
Source: Sci Rep. 2015 Feb 6;5:8289. doi: 10.1038/srep08289 (PMC4319162; doi:10.1038/srep08289)
Supplement: Supplementary Information [file srep08289-s1.pdf]

# Ultrafast Multi-Level Logic Gates with Spin-Valley Coupled Polarization Anisotropy in Monolayer $MoS_2$

Yu-Ting Wang, Chih-Wei Luo, Atsushi Yabushita, Kaung-Hsiung Wu, Takayoshi Kobayashi, Chang-Hsiao Chen and Lain-Jong Li

## Supplementary Information

## S1. Results of 2.01 eV excitation at 78 K and 1.89 eV excitation at room temperature

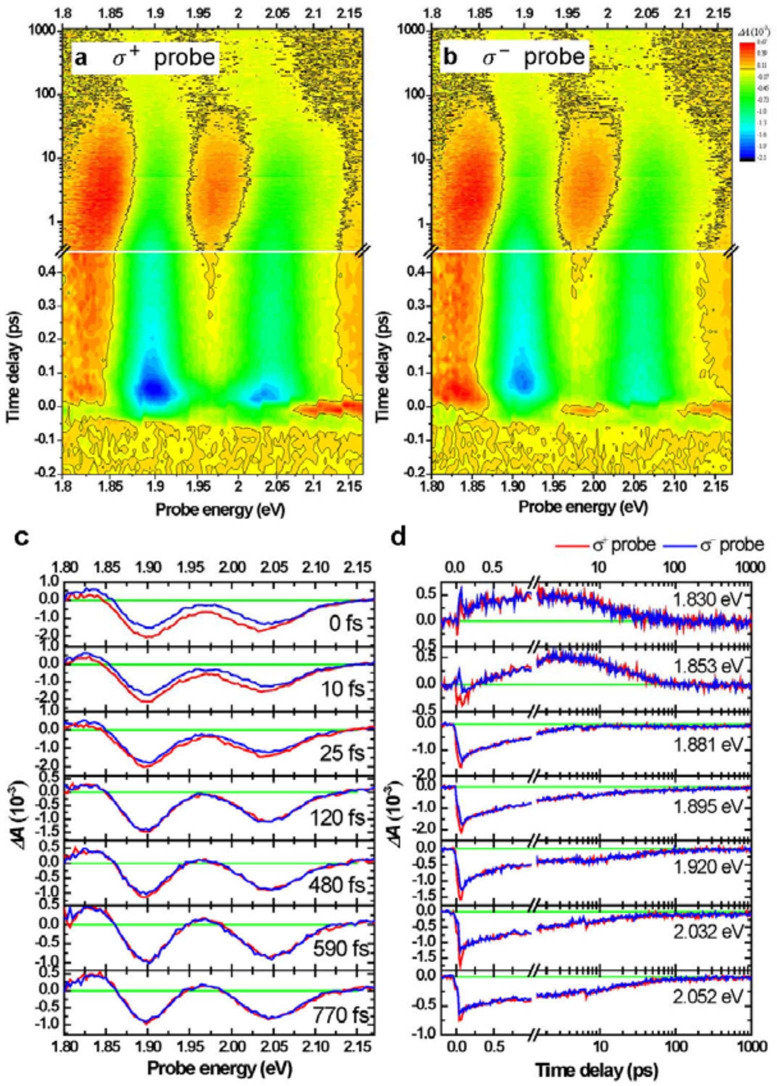


**Figure S1.** Transient difference absorbance ($\Delta A$) induced by excitation using $\sigma^+$ circularly polarized pump pulse with the photon energy of 2.01 eV and probed by (a) $\sigma^+$, (b) $\sigma^-$ circularly polarized pulse at 78 K. The black curves are contours of $\Delta A$ being zero. (c) Time-resolved $\Delta A$ spectra at various time delays between pump and probe pulses. (d) Probe delay time traces of $\Delta A$ at various probe photon energies. In (c) and (d), the red and blue lines represent $\sigma^+$ and $\sigma^-$ probe, respectively. The horizontal green lines show $\Delta A = 0$.

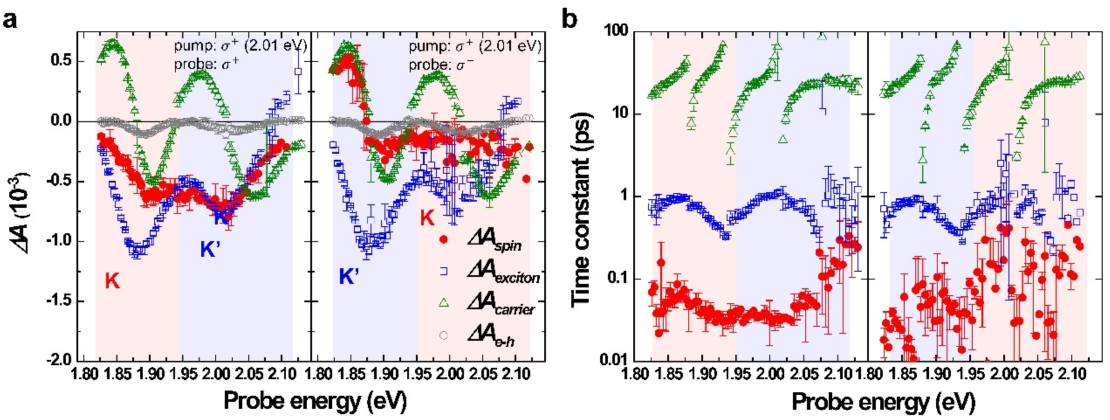


**Figure S2.** Triple exponential fitting results of the delay time traces of $\Delta A$ data excited by 2.01 eV and $\sigma^+$ pump pulse at 78 K. Left column: $\sigma^+$ probe. Right column: $\sigma^-$ probe. Solid circles (red), open squares (blue), open triangles (green), and open circles (gray) represent the components for spin randamization, exciton dissociation, hot carrier relaxation and electron-hole recombinataion, respectively. (a) $\Delta A$ spectra, (b) time constant of each component.

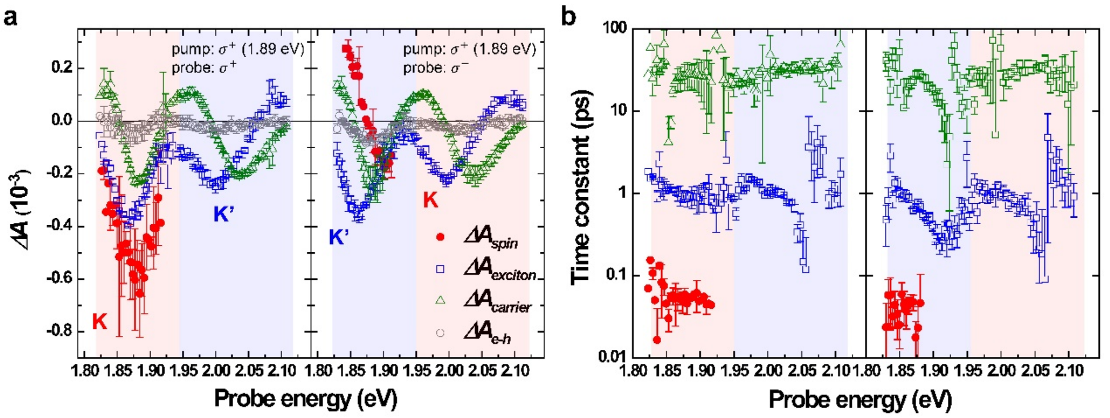


**Figure S3.** Triple exponential fitting results of the delay time traces of $\Delta A$ data excited by 1.89 eV and $\sigma^+$ pump pulse at room temperature (293 K). Left column: $\sigma^+$ probe. Right column: $\sigma^-$ probe. Solid circles (red), open squares (blue), open triangles (green), and open circles (gray) represent the components for spin randamization, exciton dissociation, hot carrier relaxation and electron-hole recombinataion, respectively. (a) $\Delta A$ spectra, (b) time constant of each component.

## S2. Estimation of time-dependent transition energy

The time-dependence of mean energy of transition A can be estimated from the first moment of photon energy of the ΔA spectrum of A band. The 0th moment and 1st moment of the ΔA spectrum corresponding to the integrated spectral area and mean photon energy of the ΔA spectrum are given as,

$$M_0(t) = \int_{\omega_i}^{\omega_f} \Delta A(\omega,\, t) d\omega,$$

$$M_1(t) = \int_{\omega_i}^{\omega_f} \frac{\Delta A(\omega,\, t)\cdot\omega}{M_0} d\omega.$$

Here $\omega_i$ and $\omega_f$ are the minimum and maximum transition energies of the corresponding band, respectively.

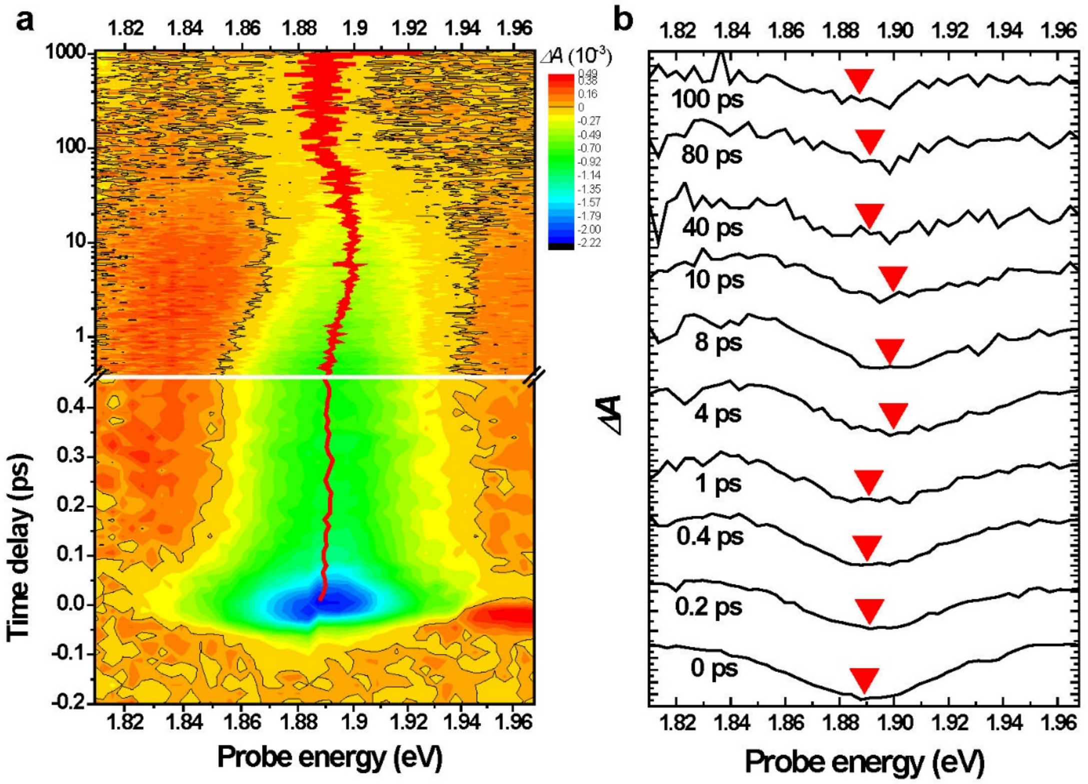


**Figure S4.** Transient difference absorbance ($\Delta A$) induced by excitation using $\sigma^+$ circularly polarized pump pulses with the photon energy of 1.89 eV and probed by $\sigma^+$ circularly polarized pulses at 78 K. (a) The mean transition energy of band A (the red line) as a function of time delays and probe photon energies. (b) Transient difference absorption spectrum at ten delay times from 0 ps to 100 ps. The red triangles show the mean transition energy at various time delays.

## S3. Dynamics of the valley population of monolayer $MoS_2$

The time dependent difference in absorbance due to population dynamics in a certain valley (K or K' valley) can be described by the following equation:

$$\Delta A_1 e^{-k_1 t} + \Delta A_2 e^{-k_2 t} + \Delta A_3(-e^{-k_2 t} + e^{-k_3 t}) + \Delta A_0$$

$\Delta A_1$:the difference absorbance due to spin randomization with the rate of $k_1$ which does not contribute to the total valley population.

$\Delta A_2$:the difference absorbance due to the loss of the K (or K') population induced by intervalley scattering at the rate of $k_2$.

$\Delta A_3$:the difference absorbance due to the increase of the K' (or K) population induced by both intervalley scattering with the rate of $k_2$ and intravalley scattering with the rate of $k_3$.

This equation should be equal to the triple exponential fitting in the main text.

$$\Delta A_1 e^{-k_1 t} + \Delta A_2{}' e^{-k_2 t} + \Delta A_3{}' e^{-k_3 t} + \Delta A_0$$

Thus,

$$\Delta A_2 = \Delta A_2' + \Delta A_3{}'$$

$$\Delta A_3 = \Delta A_3{}'$$

## S4. Transient spectroscopy measurement system

The monolayer $MoS_2$ is resonantly excited by the pump pulses from a wavelength-tunable optical parametric amplifier (OPA). A light source of the OPA is a regenerative amplifier (Legend-USP-HE; Coherent) seeded with a Ti:sapphire laser oscillator (Micra 10; Coherent). The probe pulses with a visible broadband spectrum are produced by self-phase modulation of the regenerative amplifier pulse in a sapphire disk. The polarizations of the pump and probe beams are adjusted to be $\sigma^+$ or $\sigma^-$ by the broadband $\lambda/4$ waveplates. The pump and (probe) beam are focused on the sample in a spot with the area of $1.3 \times 10^{-4}$ cm$^2$ ($0.7 \times 10^{-4}$ cm$^2$) and the pulse energy of 40 μJ (3 μJ). The time-resolution of the measuring system is estimated to be 30 fs. The transient absorbance changes of the probe pulses induced by the pump pulses are detected by a CCD camera at all probe wavelengths simultaneously. The sample is mounted inside a cryostat to control the environmental temperature of the sample.

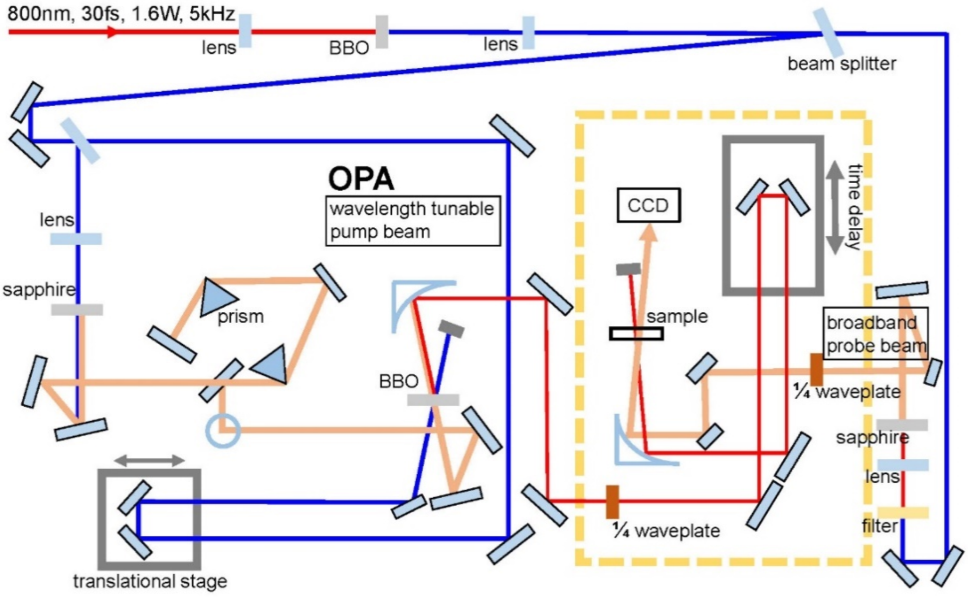


**Figure S5.** Schematic diagram of the pump/probe system. It is composed of the optical parametric amplifier (OPA) pump and the visible broadband probe. The part inside of the yellow-dashed line is the measurement system for transient absorption spectroscopy.

## S5. Sample preparation

*CVD Growth of $MoS_2$:* $MoS_2$ continuous monolayer film was synthesized based on our previous work[1]. In brief, sapphire (0001) substrates (Tera Xtal Technology Corp) were first cleaned in a $H_2SO_4/H_2O_2$ (70:30) solution heated at 100 °C for 1 hr. The substrates were placed in the center of a 1” tubular furnace on a quartz board. Precursors of 0.3 g $MoO_3$ (Sigma-Aldrich, 99.5%) in an $Al_2O_3$ crucible were placed 3 cm away from the substrates and S (Sigma-Aldrich, 99.5%) powder in a quartz tube was placed 8 cm away from the open-end of the furnace at an upstream position in a 1” quartz tube. The furnace was first heated to 150 °C at 10 °C /min rate with 80 sccm Ar at 10 Torr and annealed for 20 minutes until reaching 650 °C at 25 °C/min rate and then maintained for 10 minutes. Sulfur was heated by a heating belt at 160 °C when the furnace reached 400 °C. After growth, the furnace was slowly cooled to room temperature.

### Supplementary reference

S1. Lee, Y.-H. *et al.* Synthesis of large-area $MoS_2$ atomic layers with chemical vapor deposition. *Adv. Mater.* **24,** 2320–2325 (2012).
